# Processing different kinds of semantic relations in picture-word interference with non-masked and masked distractors

**DOI:** 10.3389/fpsyg.2014.01183

**Published:** 2014-10-20

**Authors:** Markus F. Damian, Katharina Spalek

**Affiliations:** ^1^School of Experimental Psychology, University of BristolBristol, UK; ^2^Department of German Studies and Linguistics, Humboldt-Universität zu BerlinBerlin, Germany

**Keywords:** spoken production, picture-word interference, lexical access, object naming, competition

## Abstract

Spoken production requires lexical selection, guided by the conceptual representation of the to-be-named target. Currently, the question whether lexical selection is subject to competition is hotly debated. In the picture-word interference task, manipulating the visibility of written distractor words provides important insights: clearly visible categorically related distractors cause interference whereas masked distractors induce facilitation (Finkbeiner and Caramazza, 2006). Now you see it, now you don't: On turning semantic interference into facilitation in a Stoop-like task. We explored the effect of distractor masking in more depth by investigating its interplay with different types of semantic overlap. Specifically, we contrasted categorical with associatively based relatedness. For the former, we replicated the polarity reversal in semantic effects dependent on whether distractors were masked or not. Post-experimental visibility tests showed that weak semantic facilitation with masked distractors did not depend on individual variability in participants' ability to perceive the distractors. Associatively related distractors showed facilitation with non-masked presentation, but little effect when masked. Overall, the results suggest that it is primarily distractor activation strength which determines whether semantic effects are facilitatory or interfering in PWI tasks.

## Introduction

A hotly contested issue within recent research on language production is whether accessing a word in the mental lexicon (i.e., the store of words a speaker knows) is a competitive process or not. Competition is a ubiquitous concept in various aspects of language processing (e.g., Duffy et al., 1988; MacDonald et al., 1994; Green, 1998), and many models of language production likewise assume that lexical access is accomplished via a competitive principle. Most (if not all) models of language production stipulate that word preparation involves the temporary activation of a cohort of semantic alternatives. For instance, according to the influential model by Levelt et al. (1999), a number of competitors are initially co-activated until a winner is chosen—usually the intended word, or, in case of a speech error, often a semantically related word that has accumulated most activation. However, accounts differ in whether they depict the eventual selection of the target item as competitive or not. Competition in this context implies that the time to choose a target is dependent on the number of co-activated competitors and their activation strength. Competition can be implemented either as lateral inhibition (e.g., Cutting and Ferreira, 1999) or by a rule such as Luce's choice ratio (Luce, 1959, 1986; see Roelofs, 1992) in which the time to choose a target word varies as a function of the target word's activation in relation to the activation of its competitors.

A paradigm widely used to study lexical access in spoken word production is the picture-word interference (PWI) task (first introduced by Rosinski et al., 1975): on a given trial, participants see an object which they have to name, and naming latencies are measured. At the same time or in close temporal proximity, a distractor word is presented either visually or auditorily, and participants are instructed to ignore the distractor and focus on object naming. A standard finding in PWI tasks is the *semantic interference effect* (e.g., Glaser and Düngelhoff, 1984; Schriefers et al., 1990; Damian and Martin, 1999): participants show slower average object naming latencies when distractor and target belong to the same semantic (taxonomic) category (e.g., *lion*-*monkey*) than when they are unrelated (e.g., *lion*-*cupboard*). This finding has been interpreted as evidence for competitive selection: the distractor word increases the activation of a non-target representation, thereby intensifying the underlying competition (see Roelofs, 1992, for computational modeling of this principle). However, this interpretation has recently been challenged based on a number of findings from PWI tasks which are potentially difficult to accommodate within a competitive framework, and alternative, non-competitive accounts of PWI (and more broadly, word production) have been introduced (e.g., Mahon et al., 2007; see Mulatti and Coltheart, 2012; Spalek et al., 2013, for recent overviews).

Semantic interference in PWI tasks arises most reliably when (a) distractor words are clearly visible (assuming visual distractor presentation), as opposed to when they are masked and hence difficult to see, and (b) target and distractor are coordinates of the same semantic category. In the following, we summarize the current state of knowledge with regard to these two aspects, and we then report an experiment which investigates how distractor visibility and semantic relation relate to each other.

### Distractor visibility

An important recent observation is that the semantic interference effect demonstrated in numerous previous studies reverses in polarity when distractors are masked. Finkbeiner and Caramazza (2006) compared picture-word interference with clearly visible and with masked distractors. In the latter case, participants were, according to post-experimental interviews, not consciously able to perceive the distractors. With clearly visible distractors, Finkbeiner and Caramazza obtained semantic interference effect (32 ms in their first experiment), but critically, masking of the distractors reversed the polarity of the effect such that it turned into strong and reliable facilitation (32 ms). This pattern was subsequently replicated by Dhooge and Hartsuiker (2010; Experiment 2) with Dutch speakers and materials, although resulting in somewhat smaller effects: semantic interference of 15 ms in the “visible” condition contrasted with semantic facilitation of 12 ms in the “masked” condition (presentation parameters between Dhooge and Hartsuiker's and Finkbeiner and Caramazza's studies were largely comparable).

These findings are crucial because they contribute to the wider debate on whether or not lexical retrieval in spoken word production is competitive. For advocates of a competitive view, it is not easy to explain why masking of distractors should reverse the polarity of semantic effects: without additional assumptions, their view would predict that masked distractors either generate semantic interference (perhaps reduced in size), or possibly lead to a null finding. Finkbeiner and Caramazza (2006) argued that the polarity reversal supports an account which dispenses with the notion of lexical competition altogether and instead locates semantic interference effects in PWI tasks at a post-lexical level. According to the “response exclusion hypothesis” (REH; see Mahon et al., 2007, for a detailed outline of this account), lexical access is fundamentally non-competitive, hence spreading of activation at the lexical level (which in the case of PWI arises from distractor processing) generally has facilitatory effects. In addition, however, at a later, post-lexical processing level, semantically related distractors can cause interference which may offset the facilitatory effects arising at the earlier processing levels. A distractor word in a PWI task is thought to temporarily occupy a single-channel prearticulatory “response buffer,” and needs to be removed before target naming can proceed. The time to remove a distractor word from the response buffer mainly depends on its “response relevance.” For instance, if the participant has to name a picture of an animal and the distractor word is the name of an animal (i.e., belongs to the same taxonomic category as the target), it is more difficult to purge the channel than if there is no relationship. In the same vein, if the task is to name an object, semantically related verbs do not interfere with naming, as demonstrated by Mahon et al.^1^ To account for the polarity reversal in semantic PWI effects as a result of distractor masking, Finkbeiner and Caramazza (2006) reasoned that because masking prevents the distractor word from occupying the response buffer, no interference arises. At the same time, masked distractor words are still sufficiently processed to generate semantically based facilitation in the mental lexicon.

Other explanations for the polarity reversal with masked distractor presentation in PWI tasks are possible, however, and crucially, competition need not be abandoned. Piai et al. (2012) suggested that whether or not competition arises might depend primarily on the activation strength of the distractor. Only distractors whose activation crosses a particular threshold will engage in competition with the target and generate semantic interference effects. By contrast, if distractor activation is too low, distractors will not be considered for response selection and hence will not lead to interference; however, such weakly activated distractors might still cause facilitation via overlap with the target at the semantic level. Hence, the polarity reversal demonstrated by Finkbeiner and Caramazza (2006) and Dhooge and Hartsuiker (2010) is explained not with the assumption that semantic facilitation and interference arise at two different loci (at lexical-semantic and response buffer levels, as advocated by the response exclusion hypothesis). Rather, the claim is that only strongly activated distractors will engage in competition with the target (and hence cause interference) whereas weak distractors will merely cause semantically based priming. This “competition threshold hypothesis” shifts the explanatory focus from conscious availability of the distractor identity (as in the response exclusion account) to distractor activation strength. In other words, even distractors which are clearly visible to the participant might not result in interference if they generate only weak activation.

This prediction was tested by Piai et al. (2012) in two experiments. The first experiment manipulated visibility via presence or absence of forward and backward masks around a briefly presented distractor. In a “clearly visible” condition, primes were presented for 53 ms, and following a blank period of 13 ms, the object was presented. Because distractors are not masked, this trial structure renders the distractor relatively easy to perceive. In a “poorly visible” condition, primes were again presented for 53 ms, but now they were preceded by a forward mask consisting of hash signs for 500 ms, and backward masked by a string of consonants for 13 ms before the object was presented (the latter condition is very similar to the masking employed in Finkbeiner and Caramazza, 2006; Dhooge and Hartsuiker, 2010). Each target was from a separate semantic category, and distractors never appeared as targets. Under these conditions, results showed a null effect for the “poorly visible” condition, and a *facilitatory* effect of 15 ms in the “clearly visible” condition. A second experiment was very similar to the first one, except that now there were four target exemplars per category, and distractors also appeared as target names. Both aspects should, according to the authors, increase co-activation of multiple entries in the lexicon. Now, results showed 17 ms interference for the “poorly visible” condition, and 13 ms interference in the “clearly visible” condition. According to the authors, these findings demonstrate that strength of distractor activation is the primary variable which determines whether semantically related distractors generate facilitation or interference. Presenting distractors only briefly generally reduces distractor strength, and masking further weakens distractor processing. Other variables (such as response set membership) further influence the degree of co-activation in the lexicon. Overall, Piai et al. suggested that polarity reversals of semantic effects in PWI do not contradict a general principle of competitive lexical access. At the same time, it is clear that the notion of a “competition threshold” represents an important modification of earlier competitive models (e.g., Roelofs, 1992; Levelt et al., 1999).

In our experiment reported below, we further explored the effects of visibility and co-activation on lexical competition. As in the previous studies, we manipulated visibility as a factor with two levels (masked vs. unmasked), but we also assessed individual differences in participants' ability to extract information from briefly presented distractors. The intention was to explicitly probe the possible relationship between conscious availability of the distractor, and the size and direction of the resulting semantic effect. Finkbeiner and Caramazza (2006) merely asked participants, following the experiment, whether they had noticed any masked distractor words, and reported that only one participant reported being able to see some letters of masked words (this participant was subsequently replaced). Dhooge and Hartsuiker (2010) carried out a more explicit test of visibility: they selected pictures and words which were closely matched to the experimental stimuli, and presented them with the same timing and masking parameters as in the actual experiment. Participants were asked to indicate whether or not they had seen the distractor and if so, to report its name or some of its letters. None of their participants were able to report information on the distractors.

In our experiment we employed a lexical decision task (LDT) as a post-experimental visibility test. Participants were shown the distractor words from the earlier PWI task centered on the screen, using the same masking parameters as in the picture-word interference test. We generated and interleaved an equal number of non-words, and on each trial, participants indicated whether or not they thought the distractor was a word of their language (the experiment was conducted in German). Results from the LDT allowed us to compute individual *d*′-scores for each participant. It should be noted that because we used the same materials in the PWI and LDT task, distractors in the LDT had already presented multiple times in the PWI phase of the experiment. For this reason, performance on the LDT might overestimate individuals' ability of having identified the distractors in the earlier PWI phase. Nevertheless, we hoped to obtain a relatively wide range of variation in individual d' scores (and as will be shown below, this was clearly the case). This allowed us to explore the relation between distractor visibility and semantic effects in PWI. If conscious availability is the primary determinant of whether a semantic effect is positive or negative, then for participants with higher visibility scores, the effect should tend toward interference, whereas in participants with lower visibility scores, it should result in facilitation. By contrast, if distractor strength is the primarily important variable, then the masking procedure should generally (and independently of conscious distractor availability) weaken activation strength, and by and large, semantic effects should be facilitatory.

In making these predictions, it is acknowledged that the competition threshold claim makes it difficult to generate precise *a priori* predictions about when semantic interference should turn into facilitation. This is because the threshold itself is not objectively defined, but rather only *post-hoc* via an experimental effect—if semantic interference is found in an PWI task, then distractors must have been strongly enough activated to cross the threshold; if not, they were not.

### Type of semantic relationship

A further facet contributing to the recent debate on lexical competition in word production concerns the type of semantic relationship between distractor and target. Interference is generally only obtained with co-hyponyms (targets and distractors belonging to the same taxonomic category); other types of semantic relationships such as part-whole relationships (Costa et al., 2005; but see Sailor and Brooks, 2014), hypernymy-hyponymy (Kuipers and La Heij, 2008; but see Hantsch et al., 2005), and semantically related nouns and verbs (Mahon et al., 2007) tend to generate facilitation. The fact that interference is restricted to categorically related distractors and targets poses potential difficulties for the competitive view: if interference in PWI arises as a result of conceptual overlap, why does interference not extend to forms of overlap other than strict category membership? The REH accounts for this pattern via a principle of “response relevance”: categorically related distractors are response relevant in the sense that they could potentially be plausible target responses, and so take more time to remove from the response buffer. Non-categorically related distractors are not response relevant and so don't result in interference in the buffer (but might generate facilitation via higher-level overlap with the target).

In the experiment reported below, we manipulated not only distractor visibility (see previous section) but also compared and contrasted the effects of categorically and associatively related distractors. We will briefly summarize previous findings on the effects of associative relationships in the PWI before outlining our motivation for including this form of relatedness in our own experiment.

Whereas taxonomic (from here onwards: categorical) relatedness between target and distractor slows down naming (e.g., Glaser and Düngelhoff, 1984; Schriefers et al., 1990; Damian and Martin, 1999), findings for associatively related items are more mixed, rendering either null results, or facilitation. Lupker (1979) compared the effects of categorical and associative relations in picture-word interference. While he found that categorical relations caused interference, he did not observe any effect of associative relations. In a second experiment, he tested if there were additive effects of categorical and associative relationships by comparing categorically related distractors with distractors that were both categorically and associatively related, but both types of distractors caused the same amount of interference. Subsequently, however, facilitatory effects of associative relationships were reported. La Heij et al. (1990) manipulated the association strength for categorical distractors. While they found interference for weakly associated categorical distractors, they did not observe any effects for strongly associated categorical distractors. This pattern was explained with the assumption that categorical overlap causes interference whereas an associative relationship generates facilitation, resulting in a null result if both types of relationship are combined. Associatively based facilitation was subsequently demonstrated more explicitly: Alario et al. (2000) contrasted the effects of categorically, non-associated distractors with those of associated, non-categorically related distractors (e.g., dog-bone). They reported interference effects for categorically related distractors and facilitatory effects for associatively related distractors (although possibly following slightly different time courses; this aspect is less relevant for present purposes). Abdel Rahman and Melinger (2007, Experiment 3) found the same pattern, with interference for categorically related distractors and facilitation for associatively related distractors.

The dissociation between associatively and categorically related distractors in PWI was recently further explored via brain imaging by de Zubicaray et al. (2013). They contrasted categorical with “thematic” relations, i.e., associations caused by a common theme (e.g., *mouse* and *cheese* being related through an “eating” event). Behaviorally, they observed facilitation from thematically related distractors, and interference from categorically related distractors, relative to an unrelated condition. In the fMRI data, both types of relationship caused deactivations in the mid portion of the left middle temporal gyrus, but categorical relations also involved the posterior left MTG, while thematic relations involved the left angular gyrus. This finding underscores the assumption that categorical and thematic relations are processed differently.

To sum up, the available evidence suggests that categorical and associative relations cause different effects and should therefore be carefully controlled in studies on picture-word interference. This, however, is not always the case, and “mixed” stimuli might at least partially account for the polarity reversal of semantic effects in PWI tasks outlined in the previous section. Potentially, the categorical relationship asserts itself more strongly in the visible condition and the associative relationship more strongly in the masked condition, making the net effect appear like a polarity reversal of the categorical effect. Note that this result would not be necessarily at odds with the response exclusion hypothesis: This account predicts that masking prevents distractors from entering a buffer, hence, masking eliminates the interference component. Unlike the explanation offered by Finkbeiner and Caramazza (2006), however, we suggest that different items might be responsible for interference in the visible condition and facilitation in the masked condition. Unfortunately, Finkbeiner and Caramazza (2006) do not provide a list of their items, but Dhooge and Hartsuiker (2010) do. Examining their items, one sees that they used both weakly associated target-distractor pairs (e.g., *spoon*-*knife*; *monkey* -*bear*) as well as strongly associated items (*lion*-*tiger*; *apple*-*pear*), and furthermore, also pairs that can be thought of as part and whole (*farm*-*shed*; *pot*-*lid*).

In order to carefully tease apart the potential influence of categorical and associative relations in both masked and visible distractor presentation, we carried out an experiment which varied both types of relatedness separately. This lead to three related experimental conditions: one in which distractors and targets were categorically but not associatively related, one in which they were associatively but not categorically related, and one in which they were both categorically and associatively related. Associative relatedness was determined with subjective ratings in a pre-study, as well as *post-hoc* via participants' ratings. If it is true that the polarity reversal is mainly due to the associative (facilitatory) component having a stronger effect with masked distractors, and the categorical (interfering) component emerging stronger with non-masked presentation, we should observe the strongest polarity reversal for the combined items. If our hypothesis is correct, the categorical relation mainly causes the interference in visible presentation and the associative relation generates the facilitation in masked presentation. For the categorically related items (without additional association), we should hence observe interference in the visible condition and a null effect in masked presentation. Finally, for associatively related items, we should see an increase of the facilitation effect in masked presentation conditions.

## Methods

### Participants

Forty-eight students (28 women) from Humboldt-University Berlin took part in the experiment and were paid for their participation. Their mean age was 25 years. All participants were native speakers of German.

### Materials

Twenty line drawings of common objects were used as targets. For each picture (e.g., *orange*), three distractor words were selected: a semantically related word (i.e., a category coordinate, e.g., *banana*), an associatively related word (i.e., a related word from a different category, e.g., *juice*), and a semantically and associatively related word (e.g., *lemon*). Distractor words in the three different conditions were matched on length and frequency. We created three corresponding unrelated conditions by recombining the related distractors within each relatedness type with different pictures. Therefore, for each of the three relatedness types (categorically related, associatively related, combined), the same pictures and words were used in both the related and the unrelated condition. Each participant saw a target word in all six conditions (three critical conditions and three control conditions). See Appendix for a list of all combinations. A different randomization was created for each participant to avoid order effects.

Strength of associative relations was established pre- and *post-hoc*. In a pre-study, we had investigated the association strengths of 22 line drawings, asking 24 participants to rate the association strength for a target word and the intended distractor word on a scale from 1 (“not related at all”) to 7 (“very strongly related”). These participants came from the same pool as those who participated in the actual experiment, but none were in the experiment. Table 1 presents the results from the pre-study. As intended, items in the associative condition were rated to be more strongly related than in the categorical condition, *t*_(21)_ = 6.85, *p* < 0.001, and similarly, items in the combined condition were rated to be more strongly related than in the categorical condition, *t*_(21)_ = 10.86, *p* < 0.001. Associative and combined conditions did not differ in association strength, *t* < 1, and none of the baseline conditions differed from each other, all *p*s > 0.20.

**Table 1:** **Association strength (means and standard deviations) from the pilot and *post-hoc* experimental ratings**.

	**Categorical**	**Associative**	**Combined**
**PILOT RATINGS**			
Related	3.96 (0.63)	5.63 (1.09)	5.55 (0.59)
Unrelated	1.45 (0.39)	1.69 (0.69)	1.60 (0.65)
***POST-HOC* RATINGS**			
Related	4.31 (0.67)	5.83 (1.18)	5.67 (0.59)
Unrelated	1.49 (0.41)	1.29 (0.20)	1.56 (0.70)

We also carried out the same rating study, using only the 20 line drawings eventually used in the experiment, after the PWI task and visibility tests (outlined below under the header “Rating study”). Table 1 presents the results from the *post-hoc* rating as well. The *post-hoc* ratings confirmed the pilot results: the associative and the combined conditions had stronger association strengths than the categorical condition, *t*_(19)_ = 5.51, *p* < 0.001 and *t*_(19)_ = 8.40, *p* < 0.001, respectively, whereas the combined and the associative conditions did not differ in their association strength, *t* < 1. The three baseline conditions did not differ significantly from one another [baseline categorical vs. baseline associative: *t*_(19)_ = 1.55, *p* = 0.07; baseline categorical vs. baseline combined: *t* < 1; baseline associative vs. baseline combined: *t*_(19)_ = 1.27, *p* = 0.11].

For the visibility assessment (lexical decision task; see below), the 60 distractors as described above were used as “word” stimuli. Sixty non-words were created by using existing words and replacing one or two letters. These letter changes could occur in any position in the word, and care was taken to change letters in each position equally often. Non-words were matched in length to the word targets. This resulted in 120 target stimuli for the LDT (60 words and 60 non-words).

### Procedure and apparatus

Participants carried out three different tasks: the PWI task, a lexical decision task and a rating task. Within the PWI task, the order of the blocks corresponding to presentation mode (non-masked vs. masked) was counterbalanced across participants. An entire testing session lasted about an hour. PWI and lexical decision tasks were programmed and run with Presentation (NeuroBehavioral Systems). The rating task was carried out with Excel from Microsoft Office. All tasks were presented on a 19” CRT monitor with a refresh rate of 75 Hz (13.33 ms).

#### Picture-word interference task

Participants were instructed to name objects presented on the computer monitor as quickly and accurately as possible. Trial timing and masking procedure were adopted from Finkbeiner and Caramazza's (2006) work, as follows: in the *non-masked presentation mode*, a trial started with a fixation cross that was presented for 500 ms in the center of the screen. The distractor word was presented centered on the screen for 53 ms (4 screen refresh cycles). Picture and word were then presented together for 2000 ms. Participants' responses triggered a voice key, and latencies were measured relative to picture onset. In the *masked presentation mode*, a trial started with a forward mask (##########) presented for 500 ms. The word was presented centered on the screen for 53 ms. It was replaced by the picture and a non-pronounceable mask consisting of a string of 10 consonants presented in the same location as the distractor word. Picture and mask were presented together for 2000 ms. Participants' responses triggered a voice key, and latencies were measured relative to picture onset. The use of a consonant string as a backward mask was motivated by Finkbeiner and Caramazza (2006) who refer to findings having shown its particular effectiveness in eliminating phonological priming effects.

#### Lexical decision task

Because the aim was to assess visibility of distractors in the masked presention mode of the PWI task, the trial structure was chosen to be very similar. A forward mask (##########) was presented for 500 ms centered on the screen, followed by a letter string presented for 53 ms. The letter string was replaced by a backward mask consisting of a string of 10 consonants presented in the same location as the distractor word. The mask stayed in place until the participant had made a response. Participants were instructed to decide whether or not the briefly presented string was an existing word of their language. They were encouraged to make a guess if they felt they had not seen a stimulus at all. The 120 target stimuli (60 words and 60 non-words) were randomly intermixed, with a new sequence for each participant.

#### Rating study

The names of the 20 target pictures and their related distractors were presented as pairs. For each pair, participants were instructed to indicate how strong the association between the two concepts was, using a scale from 1 (“not related”) to 7 (“strongly related”). Items were divided into six blocks, with a given target word occurring only once per block. Each relatedness condition (categorical, associative, combined) and their respective baselines occurred equally often within a given block; the assignment of a particular item in a given condition to a block was counterbalanced across lists. Six different randomizations were created.

## Results

### Picture-word interference task

Fifty-three observations (0.5% of the data) had to be removed due to script errors. Latencies on trials with errors (4.8%) as well as latencies that differed more than three standard deviations from a participant's conditional mean (1.1%) were excluded from the analysis. Table 2 presents mean reaction times and error percentages, split by presentation mode, relatedness and type of relatedness.

**Table 2:** **Reaction times (in milliseconds) and errors (in percent) by presentation mode (visible vs. masked distractor presentation), relatedness (related vs. unrelated), and type of relatedness (categorical, associative, combined)**.

	**Non-masked distractors**			**Masked distractors**		
	**Categorical**	**Associative**	**Combined**	**Categorical**	**Associative**	**Combined**
**REACTION TIMES**						
Related	711 (132)	672 (119)	702 (77)	654 (77)	662 (78)	660 (78)
Unrelated	673 (116)	687 (125)	677 (80)	665 (80)	660 (89)	656 (86)
Difference	38	−15	25	−11	2	4
**ERRORS**						
Related	5.4 (5.4)	5.5 (5.7)	7.0 (5.4)	4.2 (4.9)	3.2 (4.4)	4.8 (5.3)
Unrelated	5.3 (6.8)	5.6 (6.0)	5.4 (6.8)	3.8 (3.8)	4.3 (4.3)	3.1 (4.1)
Difference	−0.1	0.1	1.6	0.4	−1.1	1.7

*Standard deviations in parentheses*.

#### Reaction times

Latencies were analyzed with analyses of variance (ANOVAs), with the factors presentation mode (non-masked vs. masked), relatedness (related vs. unrelated), and type of relatedness (categorical, associative, combined). The main effect of presentation mode was significant, *F*_1(1, 47)_ = 7.28, *MSE* = 14, 713, *p* = 0.010; *F*_2(1, 19)_ = 49.44, *MSE* = 782, *p* < 0.001, with 29 ms faster reaction times for the masked than the non-masked condition. The effect of relatedness was also significant, with 13 ms slower reaction times for related than for unrelated items, *F*_1(1, 47)_ = 5.70, *MSE* = 1248, *p* = 0.021, *F*_2(1, 19)_ = 5.08, *MSE* = 625 *p* = 0.036. The main effect of type of relatedness was not significant, *F*_1_ = 1.22, *p* = 3.01; *F*_2_ = 1.13, *p* = 0.333. The main effects were qualified by a significant interaction of relatedness by presentation mode, *F*_1(1, 47)_ = 7.97, *MSE* = 1421, *p* = 0.007; *F*_2(1, 19)_ = 9.34, *MSE* = 431, *p* = 0.006, an interaction of type of relatedness by presentation mode, *F*_1(2, 94)_ = 3.88, *MSE* = 774, *p* = 0.024; *F*_2(2, 38)_ = 3.64, *MSE* = 383, *p* = 0.040, and an interaction of relatedness by type of relatedness [*F*_1(2, 94)_ = 6.57, *MSE* = 1020, *p* = 0.002; *F*_2(2, 38)_ = 3.93, *MSE* = 734, *p* = 0.028]. The three-way interaction of presentation mode, relatedness, and type of relatedness was also significant, *F*_1(2, 94)_ = 10.97, *MSE* = 1160, *p* < 0.001, *F*_2(2, 38)_ = 11.15, *MSE* = 480, *p* < 0.001.

In order to further investigate the significant three-way interaction between presentation mode, relatedness, and type of relatedness, we conducted two additional analyses, as outlined below.

***Simple effects of presentation mode***. First, we investigated effects of relatedness and type of relatedness for each level of presentation mode (non-masked, masked) separately, an analysis which highlights the overall effects of distractor presentation mode on relatedness effects.

For the *non-masked presentation mode*, there was a main effect of relatedness, with slower reaction times for related than for unrelated trials, *F*_1(1, 47)_ = 11.04, *MSE* = 1649, *p* = 0.002; *F*_2(1, 19)_ = 10.74, *MSE* = 669, *p* = 0.004, an effect of type of relatedness which was significant by participants, but only marginally so by items, [*F*_1(2, 94)_ = 3.99, *MSE* = 1046, *p* = 0.022; *F*_2(2, 38)_ = 2.82, *MSE* = 795, *p* = 0. 072], and a significant interaction of relatedness and type of relatedness, *F*_1(2, 94)_ = 13.71, *MSE* = 1302, *p* < 0.001; *F*_2(2, 38)_ = 7.64, *MSE* = 992, *p* = 0.002. We further explored the interaction of relatedness and type of relatedness via paired *t*-tests. The 38 ms interference effect for categorically related items was significant, *t*_1(47)_ = 5.34, *p* < 0. 001; *t*_2(19)_ = 4.66, *p* < 0.001; 95% CI [24, 52]. The 15 ms facilitation effect for associatively related items was significant by participants only, *t*_1(47)_ = 2.31, *p* = 0.025; *t*_2(19)_ = 1.65, *p* = 0.116; 95% CI [2, 27]. The 25 ms interference effect for combined items was significant, *t*_1(47)_ = 2.64, *p* = 0.011; *t*_2(19)_ = 2.12, *p* = 0.047; 95% CI [6, 43].

For the *masked presentation mode*, neither relatedness nor type of relatedness was significant, *F*_1_ and *F*_2_ < 1. The interaction between relatedness and type of relatedness was not significant by participants, *F*_1(2, 94)_ = 1.80, *MSE* = 878, *p* = 0.172, and marginally significant by items, *F*_2(2, 38)_ = 2.96, *MSE* = 222, *p* = 0.064.

***Simple effects of type of relatedness***. Second, we focused on the variable type of relatedness, and investigated for each level (categorical, associative, combined) separately whether presentation mode (non-masked, masked) affected relatedness effects. This analysis specifically aims to identify potential polarity reversals in relatedness effects, as suggested by Finkbeiner and Caramazza (2006) and Dhooge and Hartsuiker (2010).

For *categorically related* items, the effect of presentation mode was significant, *F*_1(1, 47)_ = 9.64, *MSE* = 4539, *p* = 0.003; *F*_2(1, 19)_ = 51.61, *MSE* = 295, *p* < 0.001, and so was the effect of relatedness, *F*_1(1, 47)_ = 7.99, *MSE* = 823, *p* = 0.007; *F*_2(1, 19)_ = 6.73, *MSE* = 430, *p* = 0.018. Mode and relatedness interacted with each other, *F*_1(1, 47)_ = 30.25, *MSE* = 670, *p* < 0.001; *F*_2(1, 19)_ = 30.42, *MSE* = 274, *p* < 0.001. Paired *t*-tests showed the highly significant interference effect of 38 ms for non-masked distractors already reported in the previous section, *t*_1(47)_ = 5.34, *p* < 0. 001; *t*_2(19)_ = 4.66, *p* < 0.001; 95% CI [24, 52]. The 11 ms facilitation effect for masked distractors was marginally significant, *t*_1(47)_ = 1.91, *p* = 0.063, *t*_2(19)_ = 1.79, *p* = 0.090; *t*_2(19)_ = 1.86, *p* = 0.078; 95% CI [1, 23].

For *associated* items, the effect of presentation mode was marginally significant, *F*_1(1, 47)_ = 2.95, *MSE* = 4801, *p* = 0.092; *F*_2(1, 19)_ = 8.90, *MSE* = 511, *p* = 0.008. Relatedness was not significant, *F*_1_ = 2.17, *p* = 0.148; *F*_2_ = 1.35, *p* = 0.259, nor was the mode x relatedness interaction, *F*_1_ = 1.79, *p* = 0.188; *F*_2_ = 1.60, *p* = 0.221.

For *combined* items, the effect of presentation mode was significant, *F*_1(1, 47)_ = 8.12, *MSE* = 3985, *p* = 0.006; *F*_2(1, 19)_ = 20.98, *MSE* = 605, *p* < 0.001. The effect of relatedness was significant by participants, *F*_1(1, 47)_ = 5.81, *MSE* = 1341, *p* = 0.020, and marginally significant by items, *F*_2(1, 19)_ = 3.56, *MSE* = 875, *p* = 0.075. The mode x relatedness interaction was not significant, *F*_1_ = 1.80, *p* = 0.186; *F*_2_ < 1, *p* = 0.344.

#### Error rates

Error scores are shown in Table 2, and were submitted to logistic regression analysis with the factors presentation mode (non-masked vs. masked), relatedness (related vs. unrelated), and type of relatedness (categorical, associative, combined). The results showed a significant effect of presentation mode, *Wald Z* = 2.52, *p* = 0.012, with 1.8% more errors in the non-masked than the masked condition. Furthermore, the interaction between relatedness and type of relatedness was significant, *Wald Z* = −2.16, *p* = 0.031. Simple effects analysis showed no effect of relatedness for the “categorical” and “associative” conditions, *Wald Z* = −0.24, *p* = 0.811, and *Wald Z* = 0.69, *p* = 0.491 respectively, but a significant effect for the “combined” condition, *Wald Z* = 2.27, *p* = 0.024, with 1.6% more errors in the related than the unrelated condition. All other main effects or interactions were not significant, *Wald Z* ≤ 1.73, *p* ≥ 0.083.

### Interim summary

Overall, the latency results from the “non-masked” presentation mode replicated an existing pattern in previous research: a strong categorical semantic interference effect contrasted with a weaker associative facilitation effect. The combined effect of categorical and associative relatedness was almost perfectly additive. In the “masked” presentation mode, effects were much weaker. Most relevant is the 11 ms facilitatory effect in the categorically related condition, which compares with parallel effects in previous research of 32 ms (in Finkbeiner and Caramazza, 2006, Experiment 1) and 12 ms (in Dhooge and Hartsuiker, 2010, Experiment 2). This effect just failed to reach conventional significance (see Section Lexical Decision Task below for further analysis) but numerically, the polarity reversal of the semantic effect dependent on presence or absence of distractor masking which was highlighted by the earlier studies also emerges in the present study. In the associatively and combined relatedness conditions, very little effects emerge under masked conditions.

One possible reason why the masked effects are so small is that the masking procedure may have been too efficient, eliminating (or substantially reducing) distractor processing. The results from the post-experimental visibility test reported in the following section allow some insight into this issue.

### Lexical decision task

Table 3 summarizes the accuracy results from the lexical decision task. Overall, 71.3% of the masked words were correctly recognized, with an overall false alarm rate (i.e., “word” responses to non-words) of 33.8%. For each participant, we calculated a *d-prime* (*d*′) score based on the hits and false alarm rates for words, using the formula for R suggested by Pallier (2002). *D*′ scores ranged from 0.25 to 2.93, with a mean of 1.25 and a standard deviation of 0.67, and differed significantly from zero, *t*_(46)_ = 12.80, *p* < 0.001. This implies that the masking procedure did not fully prevent distractor visibility.

**Table 3:** **Accuracy of lexical decision task, by condition (categorical, associative, combined)**.

**Condition**	**Correctly recognized**
Categorical	70.9% (17%)
Associative	68.8% (18%)
Combined	74.3% (17%)

*Standard deviation in parentheses*.

The latter result may seem surprising, given that we chose our masking procedure to be very similar (in terms of prime durations, nature of mask, etc.) to those used by Finkbeiner and Caramazza (2006) and Dhooge and Hartsuiker (2010). Finkbeiner and Caramazza did not include formal visibility assessments in their study so it is difficult to assess whether their masking had been more stringent than ours. Dhooge and Hartsuiker included, in their second experiment, a visibility test consisting of presence/absence judgments on masked prime, but merely reported that “no distractors were reported” (p. 884). It is worth noting (see our point in the Introduction) that our visibility test possibly overestimated participants' true ability to access distractor identity in the main experiment. Nevertheless, it is clear from the lexical decision results that distractors were not perfectly masked in our study. *D*′ scores computed for each participant showed substantial variability, with some participants essentially unable to identify the distractors (those with a *d*′ close to zero) and others evidently finding it quite easy (those with the highest *d*′ scores).

The high variability in prime visibility in our study offers a possible explanation for the weak masked effects. According to Finkbeiner and Caramazza (2006), visible distractors will cause interference whereas masked and therefore unconsciously processed distractors will generate semantic facilitation. Perhaps the less-than-perfect masking in our experiment and the associated variability in individual *d*′ scores (see above) resulted in participants with good visibility generating interference whereas those with poor visibility caused facilitation. If so, the direction of the semantic effect for a particular participant should be predictable based that participant's ability to see distractors in our post-test. Note that Piai et al. (2012) competition threshold hypothesis by contrast stipulates that masked primes, independently of how well they can be perceived by an individual, should generally create only weak activation which is rarely powerful enough to cross the threshold to engage in competition with target name retrieval. Hence, the masked semantic effect in our study should be independent of variability in distractor visibility, as indicated by *d*′.

**Figure 1:**
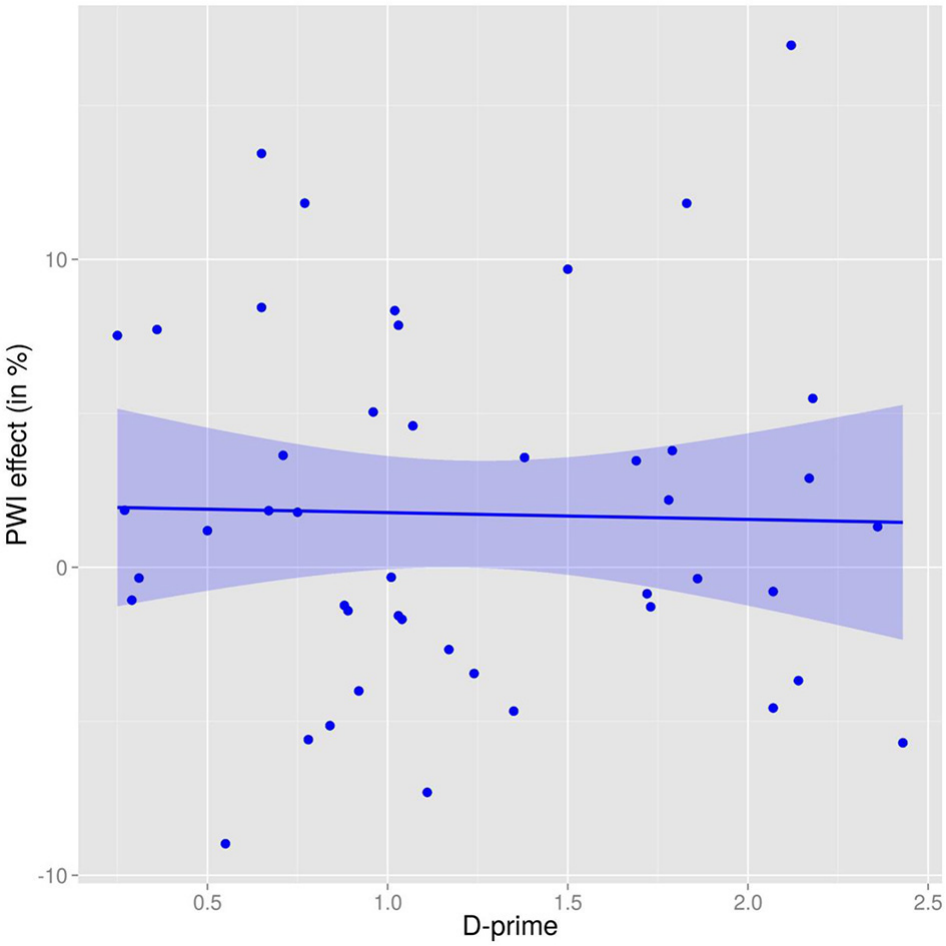
**Picture word interference effect (masked, for “categorically related” distractors; as percent relative to unrelated baseline) dependent on d-prime in lexical decision task**. Dots represent individual participants.

To investigate this issue, we focused on the “categorically related” condition (predictions for the other two types of relatedness are more difficult, as net results might be a combination of interference and facilitation). Figure 1 shows the masked categorical effect, conceptualized as a percentage change relative to the unrelated baseline condition, and dependent on *d*′ scores (dots represent individual participants). As can be seen, *d*′ scores are relatively uniformly distributed within the range, and there is no evident relationship between the experimental effect and individual visibility. A linear regression showed very little effect, *R*^2^ = 0.016, β = −0.13, *SE* = 0.15, *F*_(1, 45)_ < 1, *p* = 0.396. In other words, participants with low and high ability to consciously perceive the masked distractors showed very similar experimental effects.

Nevertheless, it is worth noting that the participant with the highest *d*′ score (2.93) showed the largest semantic interference effect (of −10%, or −56 ms; this participant is in the lower right corner of the Figure). Possibly, this participant experienced particularly good visibility of masked distractors in the experiment, which resulted in a correspondingly large interference effect. When this participant was excluded from the analysis as a potential outlier, the overall masked categorical *facilitation* effect rose to 13 ms (cf. Dhooge and Hartsuiker's, 2010, 12 ms effect in the equivalent condition), and was now statistically significant, *t*_1(45)_ = 2.19, *p* = 0.034; *t*_2(19)_ = 2.11, *p* = 0.048. A linear regression between the categorical effect and individual d-primes, again with this participant excluded, now resulted in an almost perfectly flat trend line, *R*^2^ < 0.001, β = −0.02, *SE* = 0.15, *F*_(1, 44)_ < 1, *p* = 0.874.

We conclude that despite considerable variability in participants' ability to consciously perceive the masked distractors, categorical relatedness effects in our experiment are clearly not dependent on visibility.

### Response distribution latencies

In picture-word interference tasks, mean latencies are generally shorter in masked than in non-masked conditions (Finkbeiner and Caramazza, 2006; Dhooge and Hartsuiker, 2010; Piai et al., 2012). This was also the case in our experiment, reflected in a highly significant main effect of presentation mode. Piai et al. (2012, p. 621) put forward the following line of reasoning: It is plausible to assume that, given that participants are faster under masked conditions, the shortest latencies within the response time distribution should reflect those trials on which the masking procedure was effective, whereas the longer RTs are those in which distractors are not well masked. If so, conditional means of the masked condition might represent a mixture of trials, with the shortest RTs showing facilitation and the longest ones exhibiting interference (and an overall weak effect, as was found in our experiment). We investigated this possibility via computation of Vincentized cumulative distribution curves (Ratcliff, 1979): for each participant and condition, rank-ordered latencies were divided into 20% quantiles, and mean latencies were computed for each quintile. These were then averaged across participants, which preserves the shapes of individuals' latency distributions (cf. Woodworth and Schlosberg, 1954). An analysis of this type provides information about the degree of uniformity with which an effect affects the spectrum of response latencies.

**Figure 2:**
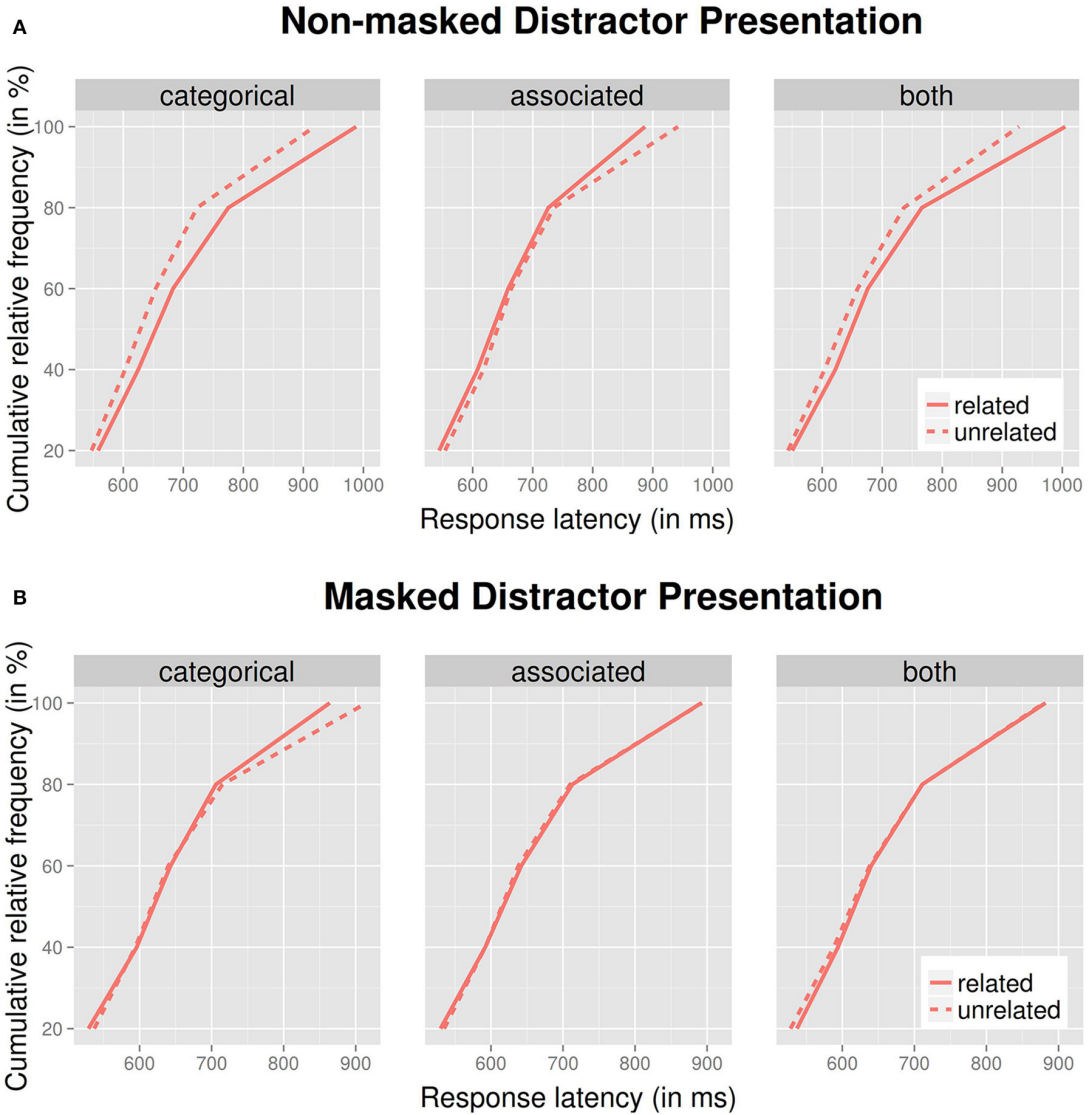
**Vincentized cumulative distribution curves for non-masked and masked distractor presentations (**A** and **B**, respectively), by relatedness type (categorical, associated, both) and relatedness (related vs. unrelated)**.

Figure 2A shows distribution curves for the “non-masked” presentation mode, and for all three types of relatedness (note that untrimmed latencies were used to generate Figure 2; see Heathcote et al., 1991). As expected from previous research (e.g., Roelofs, 2008), effects were spread out across the entire spectrum for the “categorical” and “combined” condition. The facilitatory effect for the “associated” condition emerged to a larger extent in the slower quintile. Figure 2B shows curves for the “masked” presentation mode. Intriguingly, the semantic facilitation effect weakly present in the means (cf. Table 2) predominantly emerged in the slowest (rightmost) quintile. This is clearly contrary to what one might predict on the assumption that well-masked (and hence fast) RTs should exhibit facilitation whereas poorly masked RTs show interference.

**Figure 3:**
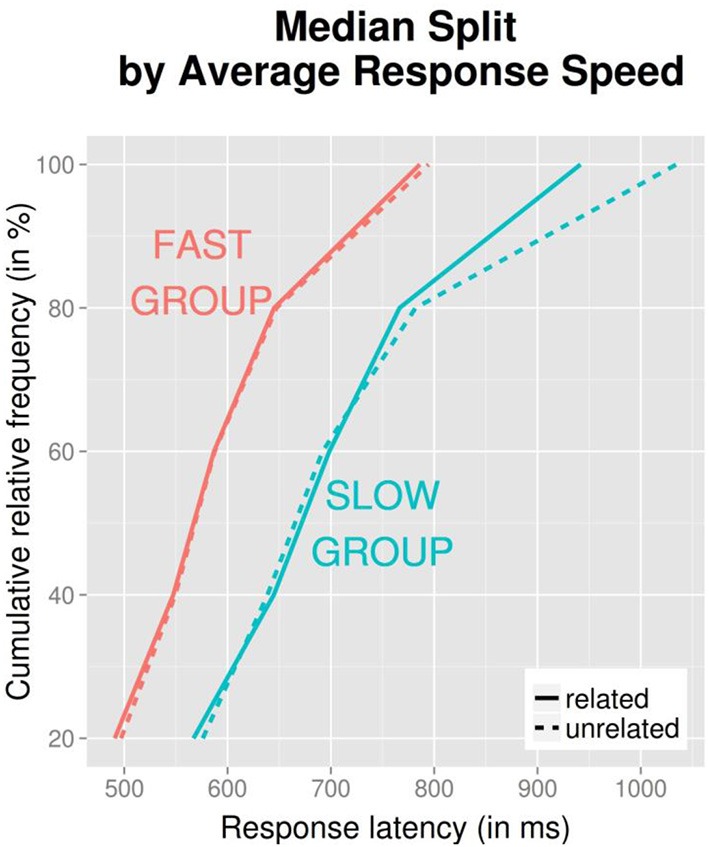
**Vincentized cumulative distribution curves for masked distractor presentation and categorically related condition, by relatedness (related vs. unrelated) and median split of participants (fast vs. slow) based on average latency in the “masked” condition**.

The manner in which Vincentiles are typically computed (for each participant individually, and then averaged) means that each participant equally contributes to all quantiles. Hence, the shown values for, say, the rightmost (slowest) quintile shown in Figure 2 represent the average of all participants' slowest quintile. Assume a scenario in which a subset of participants had better distractor visibility than others, resulting in slower latencies and semantic interference, whereas a different subset had poor visibility and hence showed faster latencies and semantic facilitation. Because in Figure 2 all participants are equally represented, this should emerge as an effect spread out across the spectrum (or perhaps no effect at all), but clearly not what is evident in Figure 2 (an asymmetry). A remaining possibility is that slower subjects, and for those individuals, slower latencies, carried the semantic facilitation effect. To look at this possibility, we performed a median split of participants into a “fast” and “slow” group, based on average latencies in the “masked” condition, and computed quintiles for the categorically related condition for each group. Figure 3 shows the results. Indeed, it appears that the semantic facilitation effect predominately stems from slower participants (and within the “slow” group, from the slowest quintile).

Given the considerable individual variability in participants' ability to recognize masked primes (see Section Lexical Decision Task), could it be that visibility is associated with slow latencies? In other words, is there an association between overall response speed and prime visibility (perhaps because prime processing slows participants down)? A further analysis suggested that this was not the case: a linear regression between overall response speed in the masked presentation mode and prime visibility as assessed by *d*′ showed no such association, *R*^2^ = 0.002, β = 0.04, *SE* = 0.15, *F*_(1, 45)_ < 1, *p* = 0.747.

## Discussion

Recent studies (Finkbeiner and Caramazza, 2006; Dhooge and Hartsuiker, 2010) have suggested that the “classic” semantic interference effect found in numerous picture-word experiments reverses into a facilitatory effect when distractors are masked such that visibility is impaired. This “polarity reversal” has been interpreted as evidence for the “response exclusion hypothesis” according to which semantic interference effects do not reflect, as commonly assumed, lexical competition between target name and distractor, but rather arise at a post-lexical response buffer level. Masking of distractors presumably prevents them from occupying the response buffer and hence from generating semantic interference. At the same time, masking still allows for some unconscious distractor processing, resulting in conceptually based facilitation. However, other interpretations of the polarity reversal pattern are possible (Piai et al., 2012): perhaps competition only takes place when potential competitors are strongly enough activated (i.e., cross a “competition threshold”). If so, masking, rather than rendering distractors unconscious, simply renders them too weak to engage in competition with target retrieval.

We report an experiment which aimed to contribute to the debate in the following way. Related or unrelated distractor words were either presented such that they were easy to identify, or masked such that they were more difficult to perceive. We additionally manipulated the type of relatedness between distractor and target: they could be either categorically related, associated, or categorically as well as associatively related. Our reasoning was that existing studies may have mixed different types of relatedness, and that semantic interference (with non-masked distractors) and facilitation (with masked distractors) might have arisen from different sets of items, namely categorically and associated pairs, respectively. If so, then the relatedness effects dependent on type of relatedness should emerge differentially with non-masked and masked presentations, and pairs which are categorically as well as associatively related should show the strongest polarity reversal. Furthermore, we added a post-experimental visibility test which allowed some insight into individuals' differential ability to perceive masked distractor words. According to Dhooge and Hartsuiker (2010) and Finkbeiner and Caramazza (2006), the directionality of semantic effects should primarily depend on distractor visibility (only visible distractors should be able to enter the “response buffer” and generate interference; invisible distractors should result in facilitation). By contrast, according to Piai et al. (2012) competition threshold hypothesis, masked distractors should largely evoke only weak distractor processing, hence semantic interference effects should generally not induce interference except under certain circumstances (see Piai et al. for details).

For distractors which were presented non-masked and were hence clearly visible to participants, our results showed substantial categorical interference (38 ms), as well as facilitation effect arising from associative relatedness (15 ms). This pattern is generally in line with previous studies on the effects of categorical vs. associative relatedness in PWI tasks (e.g., La Heij et al., 1990; Alario et al., 2000). For distractors which were categorically as well as associatively related, we found an almost perfectly additive pattern, with an empirical interference effect of 25 ms which deviated only 2 ms from the prediction based on additivity. Statistical additivity might imply, based on “additive factors logic” (Sternberg, 1969), that the two effects arise at different processing levels. This is indeed a possibility in line with previous claims. For instance, Cutting and Ferreira (1999) postulated a cascaded model of spoken word production in which phonological word forms are linked to each other via associative links. The broader claim is that lexical entries, at a sub-semantic level, might be organized according to associative relationships (e.g., Fodor, 1983; Shelton and Martin, 1992), perhaps representing co-occurrence in natural discourse (Spence and Owens, 1990). A theoretical model in which semantic interference in PWI reflects lexical-semantic competition whereas associative priming arises due to interlinked word forms could account for our findings from the non-masked presentation condition.

When distractors were briefly presented and sandwiched between forward and backward masks, effects were considerably weaker. For categorically related distractors, the “polarity reversal” predicted from the earlier studies was indeed found, but semantic facilitation in the masked presentation mode was small (11 ms) and failed to reach conventional significance. Masked effects for the associative and combined conditions were not significant. These results allow us to reject the possibility—outlined above—that previous instances of “polarity reversal” may have arisen due to differential sets of items with different types of relationship. Specifically, the predicted strong polarity reversal effect for “combined” items was clearly not present in the current results.

Results from the post-experimental visibility test allowed some further insight into the nature of the shown effects. The overall weak effects under the masked presentation mode might be attributed to too powerful masking: if masks prevent (or largely eliminate) distractor processing, then null or only small effects would be predicted. To the contrary, results from our visibility test showed (a) an overall surprisingly high ability of individuals to recognize the masked letter strings; (b) substantial individual variability in their ability to do so. *D*′ scores ranged from 0.25 to 2.93, and as visible in Figure 1 were relatively uniformly spread out within that range. This renders it unlikely that overly strict masking may have caused the weak masked priming effects in our study. An alternative is that masking was insufficient, and indeed, the REH might predict that participants with poor visibility generate semantic facilitation whereas those with good visibility cause semantic interference, plausibly resulting in a small net effect when averaged. However, our analysis which looked at categorically based masked effects in relation to individual differences (reported in Figure 1) clearly showed that this was not the case: visibility did not seem to affect polarity, nor size, of the masked semantic effects.

Overall, we interpret these results as more in line with the “competition threshold” claim introduced by Piai et al. (2012) than the “response exclusion hypothesis” favored by Finkbeiner and Caramazza (2006) and Dhooge and Hartsuiker (2010). According to the latter, given the good distractor visibility in the masked condition for at least some (perhaps most) of our participants, distractors should have been prepared for articulation in the response buffer, and semantic interference should have arisen. The fact that Figure 1 showed no clear dependence of semantic effects on distractor visibility argues against the possibility that conscious processing of distractors is the primary prerequisite for semantic interference in PWI tasks. Piai et al.'s competition threshold can more easily accommodate our results because according to that claim, masking generally reduces activation strength of distractors, and hence independent of how good individuals are at perceiving masked distractors, the overall pattern should be semantic facilitation, or perhaps a null finding.

As highlighted in the Introduction, the competition threshold view makes it difficult to generate precise *a priori* predictions about under which circumstances semantic interference or facilitation effects in PWI tasks should be obtained. To exemplify, Figure 1 showed that the individual with the highest *d*′ score showed the strongest semantic interference effect. Perhaps for this individual, distractor visibility was high enough that on most or all of trials, distractors evoked strong enough activation to cross the competition threshold and engage in competition with picture naming. Although this is not implausible, it would clearly be preferable to be able to identify distractor strength—relative to the purported threshold—beforehand in order to generate predictions about the directionality of semantic effects.

We additionally analyzed latencies via cumulative response time distribution plots (see Figure 2), and an unexpected pattern which emerged was that the weak semantic facilitation effect in the masked condition mainly emerged for slower participants, and almost exclusively in the slowest quintile of latencies. Although it is common in experimental psychology that effects are more pronounced for slower than for faster latencies, the extreme nature of the pattern found here strikes us as unusual and not easily explained. The lack of an association between overall speed of response and visibility scores certainly argues against the possibility that the slower participants for whom the semantic facilitation effect emerged were those with particularly high distractor visibility. In research on cognitive inhibition which employed response time distribution analyses, the suggestion has been made that under some circumstances, inhibitory effects may emerge only in slow quintiles because inhibition takes some time to develop (Ridderinkhof, 2002). When applying this line of reasoning to our findings, one would have to speculate that semantic facilitation is so slow to develop that it only emerges in the slower quintiles. But given that picture naming is a conceptually driven task, this suggestion makes little sense—conceptually based effects should emerge swiftly, rather than slowly. Further research is required to resolve this issue.

Overall, our findings add to the extant literature on “polarity reversals” of semantic effects in picture-word interference tasks, and suggest that these effects are genuine and not due to uncontrolled properties of stimuli (such as type of relatedness). However, our findings suggest that visibility of the distractor *per se* is not the primary determinant of whether a semantic effect is positive or negative: visibility tests implied a wide range in individuals' ability to perceive masked distractors, yet distractor masking generally resulted in weak semantic facilitation. This pattern is more in line with the notion of a “competition threshold” according to which masking generally, and independent of visibility, generally reduces distractor activation strength such that it prevents competition between distractor and target processing. Further research should illuminate the connection between conscious visibility and distractor processing more explicitly, perhaps via studies in which distractor presentation duration is systematically manipulated, and visibility associated with each particular distractor duration is assessed. The response exclusion hypothesis would predict semantic interference only for durations under which visibility tests show conscious access to distractor identity; for shorter durations, semantic facilitation should be found (which of course would disappear with too short durations). The competition threshold account predicts no systematic relation between visibility and polarity of the semantic effects in PWI tasks.

### Conflict of interest statement

The authors declare that the research was conducted in the absence of any commercial or financial relationships that could be construed as a potential conflict of interest.

## Footnotes

^1^On a strict reading of the response exclusion hypothesis, the assumption that target naming is delayed until the distractor has been removed from the response buffer predicts that latencies should exclusively depend on distractor processing, and any effects associated with target processing should be obliterated (Mulatti and Coltheart, 2012). For instance, the well-documented frequency effect in object naming (e.g., Jescheniak and Levelt, 1994) should disappear in a PWI context because processing of both high- and low-frequency target names is delayed until the distractor has been purged; however, that is clearly not the case (e.g., Miozzo and Caramazza, 2003, Experiment 1). One could therefore argue, as Mulatti and Coltheart do, that the REH has already been refuted and no further experimentation is necessary to resolve the issue.

## Appendix

Materials used in Experiment.

**Table:**

**Target picture**	**Categorical distractor**	**Associative distractor**	**Combined distractor**
Apfel (apple)	Birne (pear)	Traube (grape)	Kern (seed)
Bein (leg)	Arm (arm)	Kopf (head)	Hose (trousers)
Biene (bee)	Wespe (wasp)	Fliege (fly)	Honig (honey)
Esel (donkey)	Ochse (oxen)	Pferd (horse)	Knüppel (club)
Fluss (river)	Meer (sea)	Teich (pond)	Angler (fisher)
Fuß (foot)	Hand (hand)	Knie (knee)	Schuh (shoe)
Herz (heart)	Lunge (lung)	Darm (bowel)	Seele (soul)
Huhn (chicken)	Gans (goose)	Schwan (swan)	Ei (egg)
Kuh (cow)	Schwein (pig)	Reh (doe)	Milch (milk)
Löwe (lion)	Tiger (tiger)	Affe (monkey)	Mähne (mane)
Maus (mouse)	Katze (cat)	Hund (dog)	Käse (cheese)
Mond (moon)	Stern (star)	Komet (comet)	Nacht (night)
Nase (nose)	Mund (mouth)	Stirn (forehead)	Schnupfen (cold/flu)
Ohr (ear)	Auge (eye)	Kinn (chin)	Musik (music)
Orange (orange)	Zitrone (lemon)	Banane (banana)	Saft (juice)
Schaf (sheep)	Ziege (goat)	Kamel (camel)	Wolle (wool)
Tisch (table)	Stuhl (chair)	Schrank (wardrobe)	Holz (wood)
Tomate (tomato)	Gurke (cucumber)	Zwiebel (onion)	Nudeln (pasta)
Topf (pot)	Pfanne (pan)	Schüssel (bowl)	Deckel (lid)
Trompete (trumpet)	Pauke (kettledrum)	Flöte (flute)	Schall (sound)
